# The complete mitochondrial genome of *Cladobotryum mycophilum* (Hypocreales: Sordariomycetes)

**DOI:** 10.1080/23802359.2020.1742600

**Published:** 2020-06-26

**Authors:** Cheng Chen, Jian Wang, Rongtao Fu, Xiaojuan Chen, Xuejuan Chen, Daihua Lu

**Affiliations:** Institute of Plant Protection, Sichuan Academy of Agricultural Sciences, Chengdu, Sichuan, P. R. China

**Keywords:** *Cladobotryum mycophilum*, mitogenome, phylogenetic analysis

## Abstract

*Cladobotryum mycophilum* is the causal agent of cobweb disease in many important mushroom crops. In this study, we report the complete mitochondrial genome of *C. mycophilum* for the first time. The genome is 78,729 bp long and comprises 52 protein-coding genes (PCGs), 2 ribosomal RNA genes (rRNA), and 26 transfer RNA (tRNA) genes. The nucleotide composition of *C. mycophilum* mitochondrial genome is as follows: A (38.06%), T (34.68%), C (12.19%), and G (15.07%). Phylogenetic analysis revealed that *C. mycophilum* had a close relationship with *Cladobotryum varium* from Hypocreaceae. This study provided a basis for studies of the mitochondrial evolution of Hypocreaceae.

Cobweb disease is one of the most serious diseases in mushroom cultivation, which causes important economic losses in mushroom farms (McKay et al. 1999). The causal agents of cobweb disease are several species of the genus *Cladobotryum*, including *Cladobotryum mycophilum*, *Cladobotryum varium*, *Cladobotryum dendroides*, *Cladobotryum protrusum*, and *Cladobotryum cubitense* (Wang et al. 2018; Sossah et al. 2019)*. Cladobotryum mycophilum* has been reported to cause Cobweb disease in many important mushroom crops, such as *Agaricus bisporus*, *Pleurotus eryngii*, *Flammulina velutipes*, and *Ganoderma lucidum* (Sossah et al. 2019). At present, the complete mitochondrial genome of *C. varium* (teleomorph name *Hypomyces aurantius*) has been sequenced and published (Deng et al. 2016), but other species of the genus *Cladobotryum* were not involved. The mitogenome of *C. mycophilum* reported here would provide supplementary information on the evolution and taxonomy of *Cladobotryum.*

The specimen was isolated from a *G. lucidum* fruiting body with cobweb disease in Sichuan Province, China (105.98 E; 32.20 N). The strain was identified as *C. mycophilum* and was stored in the Sichuan Academy of Agricultural Sciences (No. Cmy1). We extracted the whole genomic DNA from the mycelia by a fungal DNA Kit D3390-00 (Omega Bio-Tek, Norcross, GA). The complete mitochondrial genome of *C. mycophilum* was sequenced using an Illumina HiSeq 2500 Platform (Illumina, San Diego, CA). Genes were assembled by MITObim v1.9 (Hahn et al. 2013). We annotated the *C. mycophilum* mitogenome with the MFannot tool, combined with manual corrections. tRNAs were predicted with the tRNAscan-SE 2.0 (Lowe and Chan 2016).

The mitogenome of *C. mycophilum* was assembled as circular DNA molecules with 78,529 bp in length, with the GC content of 27.26% and the following base composition: A (38.06%), T (34.68%), G (15.07%), and C (12.19%). The mitogenome comprised 80 genes, including 52 protein-coding genes (PCGs), 26 tRNA genes and 2 ribosomal RNA genes (*rnl* and *rns*). The 52 PCGs include 14 typical genes and 38 ORFs. Among these ORFs, we found 30 ORFs located in introns and the remaining 8 ORFs were regarded as free-standing genes. The mitogenome of *C. mycophilum* was submitted to GenBank database under accession No. MT108299.

The Bayesian inference method was used for phylogenetic analysis based on the combined gene dataset (14 typical genes) using MrBayes 3.2.6 (Ronquist et al. 2012). We established the phylogenetic relationships of *C. mycophilum* and 13 species within Sordariomycetes, using *Cairneyella variabilis* of Leotiomycetes as an outgroup. We obtained a stable evolutionary tree topology (Figure 1) with all of the recovered clades well supported (BPP ≥ 0.97). The phylogenetic analysis revealed that *C. mycophilum* had a close relationship with *C. varium* and they were clustered in a sister group. The clade (*C. mycophilum + C. varium*) was closely related to the genus *Trichoderma* of Hypocereaceae.

**Figure 1.**
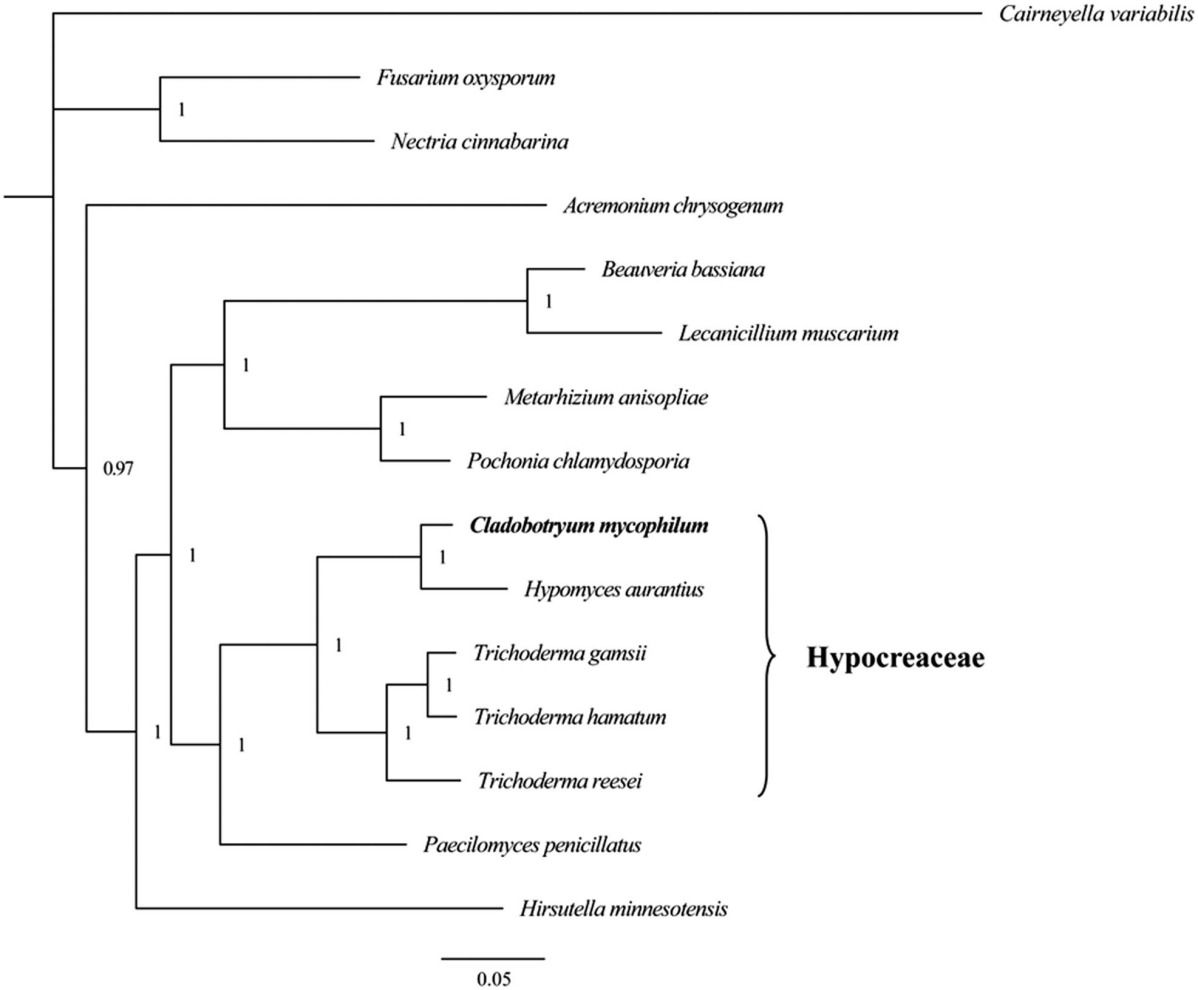
Phylogenetic relationship of 15 species based on Bayesian inference analysis of the combined mitochondrial gene set (14 typical protein-coding genes). Node support values are Bayesian posterior probabilities (BPP). Mitogenome accession numbers used in this phylogeny analysis: *Acremonium chrysogenum* (NC_023268), *Cairneyella variabilis* (NC_029759), *Nectria cinnabarina* (NC_030252), *Fusarium oxysporum* (NC_017930), *Hypomyces aurantius* (NC_030206), *Trichoderma reesei* (NC_003388), *Trichoderma hamatum* (NC_036144), *Trichoderma gamsii* (NC_030218), *Pochonia chlamydosporia* (NC_022835), *Metarhizium anisopliae* (NC_008068), *Paecilomyces penicillatus* (NC_043850), *Beauveria bassiana* (NC_010652), *Lecanicillium muscarium* (NC_004514), *Hirsutella minnesotensis* (NC_027660), *Cladobotryum mycophilum* (MT108299).
